# Pandemic Fatigue and Vaccine Hesitancy among People Who Have Recovered from COVID-19 Infection in the Post-Pandemic Era: Cross-Sectional Study in China

**DOI:** 10.3390/vaccines11101570

**Published:** 2023-10-05

**Authors:** Chenyuan Qin, Jie Deng, Min Du, Qiao Liu, Yaping Wang, Wenxin Yan, Min Liu, Jue Liu

**Affiliations:** 1 School of Public Health, Peking University, Beijing 100191, China; qincy@bjmu.edu.cn (C.Q.); 1810306145@pku.edu.cn (J.D.); dumin@bjmu.edu.cn (M.D.); yaping77@bjmu.edu.cn (Y.W.); yanwx@bjmu.edu.cn (W.Y.); liumin@bjmu.edu.cn (M.L.); 2 Institute for Global Health and Development, Peking University, Beijing 100871, China; 3 National Health Commission Key Laboratory of Reproductive Health, Peking University, Beijing 100191, China

**Keywords:** COVID-19, vaccination, pandemic fatigue, vaccine hesitancy

## Abstract

At present, the COVID-19 pandemic is still ongoing globally and the virus is constantly mutating. The herd immunity barrier established by past infections or vaccinations is gradually weakening and reinfections are occurring. To evaluate the pandemic fatigue and vaccine hesitancy among people who have recovered from COVID-19 in the post-pandemic era, we conducted an anonymous cross-sectional survey study in China from 4 July to 11 August 2023, nearly 6 months after the last large-scale nationwide infection. Basic sociodemographic characteristics, health-related factors (smoking, drinking, and chronic disease history), COVID-19 vaccination history, and self-reported long COVID were obtained as potential covariates. A series of logistic regression models were performed to examine the association between pandemic fatigue and vaccine hesitancy toward the next dose of COVID-19 vaccines via crude relative risks (cORs) and adjusted relative risks (aORs) with 95% CIs. According to our results, of the 2942 participants, 1242 (42.2%) were hesitant (unwilling or not sure) to receive the next dose of COVID-19 vaccines. The average score on the Pandemic Fatigue Scale was 21.67 ± 8.86, in which the scores of all items in the vaccine-hesitant group were significantly higher than those in the vaccine-accepting group. Additionally, the higher the pandemic fatigue level among people who have recovered from COVID-19, the more likely they were to be hesitant to receive the next dose of the COVID-19 vaccines (moderate: aOR = 2.94, 95% CI: 2.46–3.53; high: aOR = 6.88, 95% CI: 5.49–8.64). Overall, more than 40% of the recovered participants were unwilling or uncertain about the next vaccine dose, with varying degrees of pandemic fatigue. Pandemic fatigue is a potentially relevant factor for vaccine hesitancy and may hinder the translation of vaccination intention into behavior. Considering the ongoing reinfection situation, implementing a health education plan to reduce pandemic fatigue and prioritizing vaccination issues for people who have recovered from COVID-19 may be key to promoting the reduction of the COVID-19 disease burden and ensuring the health and well-being of the population.

## 1. Introduction

The coronavirus disease 2019 (COVID-19) has been raging continuously for three years, indicating a great threat to human health [1]. As of 21 August 2023, there have been over 769.8 million confirmed cases and more than 6.9 million people have died worldwide [2]. Based on the comprehensive evaluation of facts including virus variation, epidemic situation, and the basis for previous prevention and control work, China’s government successively introduced new optimization measures against COVID-19 in November 2022, trying to gradually bring people’s lives back to normal [3,4]. From late 2022 to early 2023, China experienced a nationwide Omicron variant infection, and over 80% of the whole population was estimated to be infected [3,4]. Due to high asymptomatic infection rates and limited testing capabilities, the actual number of global infections may be much higher than the reported data [2]. On 5th May 2023, the Director-General of the World Health Organization (WHO) announced that COVID-19 no longer constitutes a public health emergency of international concern (PHEIC), lifting the highest-level alert issued on 30 January 2020 in Geneva [5]. However, the COVID-19 pandemic is not yet over and has become an ongoing global health problem [5]. The evolution of SARS-CoV-2 remains uncertain [5].

Vaccination, a vital weapon to actively prevent infectious diseases, is a milestone development in the history of human civilization [6,7]. Building a herd immunity barrier and minimizing infections, severe cases, and deaths through vaccination is an important guarantee for ensuring the normal operation of society under the current situation [6,7,8]. Vaccine hesitancy is defined as refusing or delaying vaccination when available [9]. As one of the top ten health threats announced by the WHO in 2019, vaccine hesitancy still cannot be ignored in the case of large-scale infections worldwide in 2023, especially since this acquired immunity is not permanent [9,10,11]. Since previous infections do not necessarily protect individuals from reinfection, it is essential for people who have recovered from COVID-19 to prevent reinfection by receiving the next dose of COVID-19 vaccines in time. However, we seem to have overlooked the vaccine hesitancy among people with a history of COVID-19 [11]. Early studies have shown that people who have recovered from COVID-19 were more likely to be unwilling or uncertain to receive the next dose of COVID-19 vaccines than those who have not been infected, but there is a lack of recent Chinese-specific research evidence [8,12,13]. Therefore, vaccine hesitancy among this group of people in China is an important issue that deserves early research.

As the 4-year mark is approaching since the commencement of the COVID-19 pandemic (November 2019), during which a “waxing and waning” battle trajectory has been observed almost globally and affected most aspects of human life, the concept of “pandemic fatigue” has become widely recognized in both academic and popular discourse [14]. Pandemic fatigue, defined by the WHO as “distress which can result in demotivation to follow recommended protective behaviors, emerging gradually over time and affected by several emotions, experiences, and perceptions”, has been determined as an additional hurdle and risk factor to adherence to health-protective behavior by the public [15,16]. Originally developed by Lilleholt et al., the Pandemic Fatigue Scale (PFS) is a subjective questionnaire that has been used to measure subjects’ fatigue from the COVID-19 pandemic as well as from the beginning of future pandemics [17]. Many factors affect vaccine hesitancy, such as basic demographic characteristics, trust in the government, risk perception of the pandemic, knowledge of COVID-19 and vaccines, and trust in the safety and efficacy of vaccines [11,18,19,20,21]. However, few studies are focusing on the attitudes towards the pandemic after a national epidemic and its impact on the vaccination intention among recovered people in China.

At present, the COVID-19 pandemic is still ongoing globally and the virus is constantly mutating [1]. Herd immunity established by infections or vaccinations is gradually weakening, and reinfections are occurring [9,10,11]. Therefore, it is essential to understand the status and correlates of pandemic fatigue and vaccine hesitancy toward the next dose among people who have been infected before. These findings will help government authorities and relevant parties to promote future vaccination policies and strategies in an orderly and precise manner, avoid complacency, and ensure the health and well-being of the population.

## 2. Methods

### 2.1. Study Design and Participants

To evaluate pandemic fatigue and vaccine hesitancy among people who have recovered from COVID-19 in the post-pandemic era, we conducted an anonymous cross-sectional survey in China from 4 July to 11 August 2023, nearly 6 months after the last large-scale nationwide SARS-CoV-2 infection [3,4]. This online survey was performed by a professional scientific data platform (Changsha Ranxing Information Technology Co., Ltd., Changsha, China) with nearly 300 million users every month [22]. It can accurately send the electronic questionnaire to our expected representative respondents based on the clear personal information (such as age, gender, and residence) of registered members [22]. Recruitment criteria were as follows: (1) agree to fill in the questionnaire carefully; (2) ≥18 years old; and (3) complete the survey for more than 300 s. Informed consent was embedded, and all respondents provided consent for anonymized data use for academic purposes.

Based on previous survey experience [19,20,23], to obtain sufficient participants with a history of COVID-19, we used a quota sampling method based on the population proportion of provinces reported in the *Seventh National Census* to allocate a total sample size of 3000 people. The predetermined sample size was obtained using a random sampling method in each province. After excluding unqualified replies and verifying the sufficient power of the test under α as 0.05 and the confidence interval width as 0.1 p (p = hesitancy rate in this study), 2942 participants with COVID-19 infection history were ultimately included in our analysis (Table S1).

### 2.2. Pandemic Fatigue

The Pandemic Fatigue Scale (PFS) is a subjective questionnaire that has been used as a tool to measure subjects’ fatigue from the COVID-19 pandemic, which has been used cross-culturally with good validity and internal consistency [16,24]. After conducting translation into Chinese and back translation into English, we used an adapted PFS in Chinese in our questionnaire to ensure “linguistic and conceptual equivalence” [25]. The PFS comprises six items, grouped into two distinct yet highly correlated factors—behavioral and information fatigue—which both add to people’s overall experience of pandemic fatigue [16]. Participants rank their agreement with each item on a 7-point Likert scale ranging from 1 (strongly disagree) to 7 (strongly agree) [16]. All items were combined with equal weighting, and the total score ranged from 6 to 42 points. Higher scores indicate more pronounced pandemic fatigue. For ease of statistical analysis, we divided pandemic fatigue into “Low (6–18 points)”, “Moderate (19–30 points)”, and “High (31–42 points)” levels based on the total scores. In the present study, the PFS exhibited acceptable internal consistency (α coefficient = 0.791).

### 2.3. Attitude toward the Next Dose of COVID-19 Vaccines

To determine the attitude of participants with a history of COVID-19 infection towards receiving the next dose of COVID-19 vaccines, we set a question as “Are you willing to receive the next dose of COVID-19 vaccine if available?”. The answer was progressively set on a 5-point Likert scale (1 = very willing, 2 = willing, 3 = not sure, 4 = reluctant, 5 = very reluctant). We defined vaccine hesitancy as reluctance or uncertainty about receiving the next dose of COVID-19 vaccines, and then further asked for specific reasons for hesitancy.

### 2.4. Covariates

Sociodemographic characteristics (sex, age, location, education, relationship status), health-related factors (smoking, drinking, chronic disease history), and COVID-19 vaccination history were obtained as potential covariates. Risk perception items of COVID-19 reinfection were adapted from the Health Belief Model and involved two parts: perceived susceptibility and perceived severity [26]. Participants were asked “How likely do you think you are to be reinfected with COVID-19?” and “If you are reinfected with COVID-19, how severe do you think it will be?”. The 5-point scale answers (1 = very low, 2 = low, 3 = moderate, 4 = high, 5 = very high) were divided into “Low”, “Moderate”, and “High” levels. In addition, we also included self-reported long COVID symptoms and set it as a binary variable (yes or no).

### 2.5. Statistics Analysis

Statistical descriptions of all quantitative variables were reported as frequencies, percentages, means (M), and standard deviations (SD). We used the Chi-square test and *t*-test to compare the group differences among basic characteristics of vaccine hesitancy and pandemic fatigue. A series of logistic regression models were performed to examine the association between pandemic fatigue and vaccine hesitancy via crude relative risks (cORs) and adjusted relative risks (aORs) with 95% CIs. Model A was unadjusted. We controlled sociodemographic factors (gender, age, location, and education) in model B, and then plus health factors (drinking and chronic disease history) in model C. Risk perception factors (perceived susceptibility and perceived severity) were additionally added to model D. Model E adjusted for all covariates that were significantly unequally distributed across three pandemic fatigue levels. On the basis of Model E, subgroup analyses were performed among participants with different characteristics. All statistical analyses were conducted by Software SPSS 26.0 (IBM SPSS Inc., New York, NY, USA), and we set the significance level at a two-sided *p* value of <0.05.

## 3. Results

### 3.1. Characteristics of Participants

Of the 2942 participants who have recovered from COVID-19 (Table 1), 1185 (40.3%) were male, 2180 (74.1%) were ≤34 years old, 2662 (90.5%) lived in urban areas, and 2178 (74%) had at least a bachelor’s degree. Among them, a vast majority of participants were current non-smokers and only 33.6% struggled with chronic diseases. It is worth noting that less than 10% of respondents received a COVID-19 vaccination in the past six months, and 2150 (73.1%) of participants self-reported long COVID symptoms. In addition, 46.2% of all respondents believed that they had a low risk of reinfection, and more than 90% of them believed that the consequences of reinfection would not be severe.

### 3.2. Pandemic Fatigue in the Post-Pandemic Era

**Figure 1:**
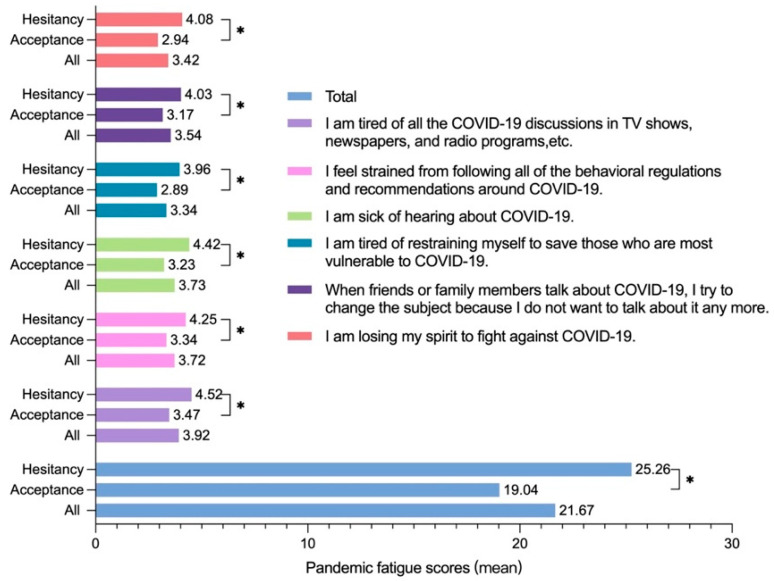
Pandemic Fatigue Scale item scores. * A *p*-value less than 0.05 is considered to be statistically significant.

As is shown in Figure 1, the average score of 2942 participants in PFS was 21.67 ± 8.86. The vaccine-hesitant group obtained a fatigue score of 25.26 ± 8.52, while that of the vaccine-accepting group was 19.04 ± 8.17. Disparities between the two groups were statistically significant (*p* < 0.05), including three information fatigue items and three behavioral fatigue items. The scores of six items in the vaccine-hesitant group were all significantly higher than those in the vaccine-accepting group.

Table 2 shows the characteristics across three pandemic fatigue groups. Only 1196 (40.7%) respondents reported a low level of pandemic fatigue. A higher level of pandemic fatigue clustered in people who are male, living in urban areas, smoking, drinking, struggling with chronic disease, higher perceived susceptibility, longer time since the most recent vaccination, and have self-reported long COVID. Moreover, this fatigue is unevenly distributed among people with different characteristics, except for relationship status and smoking history.

### 3.3. Vaccine Hesitancy toward the Next Dose of COVID-19 Vaccines

**Figure 2:**
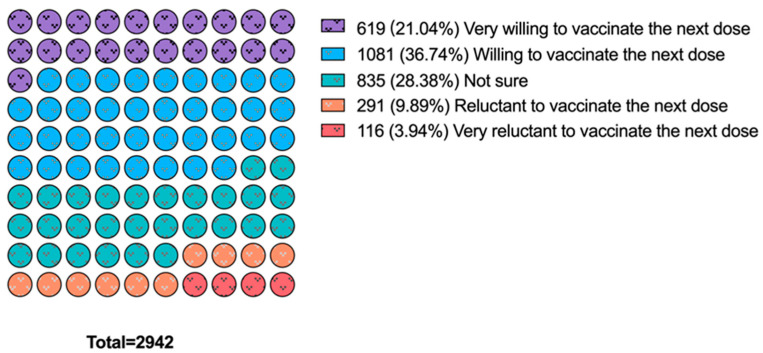
Willingness to receive the next dose of COVID-19 vaccines in recovered people (n = 2942).

Table 1 and Figure 2 demonstrate the vaccine hesitancy toward the next dose of COVID-19 vaccines. Of the 2942 participants, 1242 (42.2%) were hesitant (unwilling or not sure) to receive the next dose of COVID-19 vaccines, if available. A total of 835 (28.3%) participants marked “not sure” as the answer to the question about the intention to vaccinate against COVID-19, which accounted for 67.2% of the vaccine-hesitant group. As is shown in Table 1, among people with a history of COVID-19, those who are single, non-smokers, have low perceived severity after reinfection, did not report long COVID symptoms, and have a longer time since their last COVID-19 vaccination were more likely to be vaccine hesitant (*p* < 0.05). As the level of pandemic fatigue increased among the population, vaccine hesitancy also increased significantly. There was no significant difference in vaccination intention among people grouped by sex, age, location, education, drinking habits, chronic disease history, and perceived susceptibility to COVID-19 reinfection.

### 3.4. Association between Pandemic Fatigue and Vaccine Hesitancy toward the Next Dose of COVID-19 Vaccines

The associations between pandemic fatigue and hesitancy toward the next dose of COVID-19 vaccines among people who have recovered from COVID-19 remained stable in a series of models (Table 3). In model A, controlling for no covariates, perceived moderate (cOR = 2.89, 95% CI: 2.42–3.44) or high (cOR = 6.51, 95% CI: 5.24–8.11) pandemic fatigue among people with a history of COVID-19 infection may lead to higher vaccine hesitancy. After adjusting for sociodemographic characteristics (gender, age, location, and education) in model B, the positive association between pandemic fatigue and vaccine hesitancy remained significant (moderate: aOR = 2.91, 95% CI: 2.44–3.47; high: aOR = 6.56, 95% CI: 5.27–8.17). We obtained similar results after adding health factors (drinking and chronic disease history) in multivariable logistic regression model C. The perceived susceptibility and severity of COVID-19 reinfection, which has been shown to affect vaccination intention in previous studies [27,28], were included in model D. The results showed that people with moderate and high levels of pandemic fatigue were 2.92 (95%CI: 2.45,-3.49) times and 6.80 (95%CI: 5.44–8.50) times more hesitant to receive the next dose of vaccine than those with low levels of fatigue, respectively. Furthermore, we adjusted for all covariates that were significantly unevenly distributed in pandemic fatigue in model E, in which the higher the level of pandemic fatigue among people with a history of COVID-19 infection, the more likely they were to be unwilling to receive the next dose of the COVID-19 vaccine (moderate: aOR = 2.94, 95% CI: 2.46–3.53; high: aOR = 6.88, 95% CI: 5.49–8.64).

### 3.5. Subgroup Analyses

Subgroup analyses were exhibited in Table S2, and no modification was found in most subgroups (all *p* for interaction > 0.05), except for people stratified by education and smoking history. The association between pandemic fatigue and vaccine hesitancy weakened gradually with increasing education levels but remained significant. In the subgroup with the lowest education level, people who perceived high pandemic fatigue were 10.97 times more likely to be vaccine-hesitant than those with low pandemic fatigue, while it decreased to 3.88 times in the Master’s degree subgroup. Furthermore, the impact of pandemic fatigue on vaccination intention was more pronounced in the smoking subgroup (moderate: aOR = 3.57, 95% CI: 2.09–6.09; high: aOR = 10.47, 95% CI: 5.60–19.57) than in the non-smoking subgroup (moderate: aOR = 2.94, 95% CI: 2.42–3.57; high: aOR = 6.60, 95% CI: 5.15–8.45).

## 4. Discussion

China experienced a nationwide Omicron variant outbreak from late 2022 to early 2023 [3,4]. To our knowledge, this is the first study to explore the COVID-19 vaccination intention and pandemic fatigue among people with a history of COVID-19 nearly six months after that event. According to our results, of the 2942 participants, 1242 (42.2%) were hesitant to receive the next dose of COVID-19 vaccines, and 67.2% of the vaccine-hesitant participants were marked as “not sure”. Only 40.7% of respondents reported a low level of pandemic fatigue, and the scores of all six pandemic fatigue items in the vaccine-hesitant group were significantly higher than those in the vaccine-accepting group. Based on the results of a series of regression models, people with moderate and high levels of pandemic fatigue were more likely to be hesitant to receive the next dose of COVID-19 vaccines than those with low levels of fatigue. These findings will help government authorities and relevant parties to take the potential threat behind the pandemic fatigue and vaccine hesitancy seriously, and promote future vaccination policies among infected people in an orderly and precise manner, thus ensuring the health and well-being of the population to the greatest extent possible.

In contrast to high COVID-19 vaccine acceptance in the general population, our findings found that 42.2% of the 2942 participants were hesitant to receive the next dose of COVID-19 vaccines [27,29,30,31,32]. A growing number of studies have demonstrated that patients who have recovered from COVID-19 have lower vaccination coverage and vaccine intention than the general population [8,13,33]. The Household Pulse Survey in the United States, which collected information on vaccination status (at least one dose) and vaccination intention of 63,266 people from 21 July to 2 August 2021, found that people with previously diagnosed COVID-19 had lower vaccine coverage (aPR = 0.88, 95%CI: 0.86–0.91) [13]. In terms of willingness to be vaccinated, people with a history of COVID-19 were more likely to be unwilling to receive the next dose of vaccine than uninfected people [13]. Gerussi et al. surveyed COVID-19 vaccine hesitancy in Italian patients who had recovered from COVID-19 in 2021 and found that 34.2% and 24.9%, respectively, were undecided or unwilling to receive COVID-19 vaccines [33]. In Wuhan, China, 1422 recovered patients had a 37.8% rate of COVID-19 vaccine hesitancy, since they believed they already had enough antibodies [8]. In addition, according to our results, those who are single, non-smokers, have low perceived severity after reinfection and did not report long COVID symptoms were more likely to be vaccine hesitant. Previous studies have also confirmed the relatively lower rate of hesitancy among people with more severe illness, high perceived severity, and the perception that they may develop sequelae [8,33,34]. These findings point to the need to focus on educating and confidence-building interventions for adults at the time of COVID-19 diagnosis, at clinic visits, or hospital discharge, as well as to better educate the public about the value of vaccination.

The direct and indirect effects of COVID-19 have caused great stress to the population and led to poor mental health outcomes [35]. According to our study, more than three years after COVID-19 raged, all participants showed varying degrees of pandemic fatigue, including information fatigue and health protection-related behavioral fatigue. Compared with a survey conducted in China in early 2022 [16], the average scores of the six items of PFS in our study were significantly higher, which may be related to the different survey times and respondents, but may also imply more obvious fatigue and reduced cooperation for epidemic prevention and control at present. In this study, a higher level of pandemic fatigue clustered in people who are male, living in urban areas, smoking, drinking, struggling with chronic disease, higher perceived susceptibility, longer time since the most recent vaccination, and have self-reported long COVID. Such an unexpected, prolonged pandemic will further worsen people’s mental health and ability to cope with the situation, thus leading to mental fatigue, which is thought to be a natural psychological response of individuals through overexposure to negative information related to the pandemic and repeated implementation of behavioral restrictions [36]. On 5 May 2023, the World Health Organization declared that COVID-19 no longer constituted a PHEIC, but had transformed into an established and ongoing global health problem. Many people have generally negative attitudes toward chronic public health crises and have less motivation to engage in protective behaviors [36]. Yue et al. investigated fatigue during the three waves of the epidemic in China and showed that with the development of the epidemic, people experienced different degrees of pandemic fatigue, which may affect the occurrence of psychosomatic symptoms and perceived stress, reminding government authorities to pay attention to this phenomenon to avoid the occurrence of potential crises [36].

Across a range of models, pandemic fatigue was consistently positively associated with vaccine hesitation rates among those recovering from COVID-19, which became more significant with increasing adjustment. Previous studies in different populations have also reached similar conclusions [18,37]. Ali-Saleh et al. surveyed 2843 Arab parents and found that pandemic fatigue was indirectly associated with parents’ less positive attitudes toward vaccinating their children [37]. An anonymous cross-sectional study of general adults in Malaysia found that those with a lower pandemic fatigue score were more willing to be vaccinated against COVID-19 (OR = 2.34, 95% CI: 1.75–3.22) [18]. Pandemic fatigue is an expected and natural response to a prolonged public health crisis [18,38]. The concept of behavioral fatigue related to compliance with COVID-19 restrictions or pandemic fatigue is a social issue. During this pandemic, the impact of mental fatigue has started to have a cascading impact on vaccination efforts [39]. Fatigue may begin to cast doubt on the effectiveness of COVID-19 mitigation strategies and reluctance to take steps to end the pandemic, including vaccination [18]. Thus, pandemic fatigue is a potential correlate of vaccine acceptance and may impede the translation of vaccination intentions into behaviors. Addressing pandemic fatigue requires a robust multipronged response that addresses motivation in terms of the costs and benefits of mitigation [18].

This is a nationwide study encompassing all provinces in mainland China, which is representative and statistically efficacious. However, as described in the Methods section, this is a cross-sectional study and caution is needed when making causal inferences. Second, only those with access to the Internet were able to participate in this survey, which to some extent ignores the remaining portion of the population and may lead to selection bias. Third, we did not adjust for social media use, income, occupation, and vaccine production information when analyzing the association between pandemic fatigue and vaccine hesitancy, and there is a possibility that the level of association was misestimated. Additionally, it is important to note that this study was conducted only in patients who had recovered from COVID-19, so the levels of pandemic fatigue and vaccine hesitancy in this study were not representative of the general population. Future studies with longitudinal or experimental designs are encouraged to confirm our findings and elucidate potential mechanisms and interventions to reduce pandemic fatigue and vaccine hesitancy.

## 5. Conclusions

In this study, 42.2% of participants with a history of COVID-19 were hesitant to receive the next dose of COVID-19 vaccines, and only 40.7% of respondents reported a low level of pandemic fatigue. People with higher levels of pandemic fatigue were more likely to be hesitant to receive the next dose of COVID-19 vaccines. Therefore, given the ongoing reinfections, implementing a health education plan to reduce pandemic fatigue and prioritizing vaccination issues for people with a history of COVID-19 may be vital in promoting the reduction of the COVID-19 disease burden and ensuring the health and well-being of the population.

## Figures and Tables

**Table 1:** Characteristics and COVID-19 vaccine hesitancy among 2942 participants recovered from COVID-19 in China.

Characteristics †	Number (%)	Hesitancy Toward the Next Dose of COVID-19 Vaccine		
		n (%)	95% CI	*p* Value
Total	2942 (100)	1242 (42.2)	40.4–44.0	
Sex				0.06
Male	1185 (40.3)	525 (44.3)	41.5–47.1	
Female	1757 (59.7)	717 (40.8)	38.5–43.1	
Age (years)				0.502
<30	1390 (47.2)	575 (41.4)	38.8–44.0	
30–34	790 (26.9)	337 (42.7)	39.2–46.1	
35–39	421 (14.3)	174 (41.3)	36.7–46.1	
≥40	341 (11.6)	156 (45.7)	40.5–51.1	
Location				0.629
Urban	2662 (90.5)	1120 (42.1)	40.2–44.0	
Rural	280 (9.5)	122 (43.6)	37.9–49.4	
Education				0.960
High school and below	765 (26.0)	324 (42.4)	38.9–45.9	
Bachelor’s degree	1928 (65.5)	811 (42.1)	39.9–44.3	
Master’s degree	249 (8.5)	107 (43.0)	36.9–49.2	
Relationship status				0.001 *
Without partner	627 (21.3)	301 (48.0)	44.1–51.9	
With partner	2315 (78.7)	941 (40.6)	38.7–42.7	
Smoking				0.019 *
No	2493 (84.7)	1075 (43.1)	41.2–45.1	
Yes	449 (15.3)	167 (37.2)	32.8–41.7	
Drinking				0.488
No	1660 (56.4)	710 (42.8)	40.4–45.2	
Yes	1282 (43.6)	532 (41.5)	38.8–44.2	
Chronic disease				0.880
No	1954 (66.4)	823 (42.1)	39.9–44.3	
Yes	988 (33.6)	419 (42.4)	39.4–45.5	
Perceived susceptibility				0.056
Low	1358 (46.2)	540 (39.8)	37.2–42.4	
Moderate	1204 (40.9)	531 (44.1)	41.3–46.9	
High	380 (12.9)	171 (45.0)	40.1–50.0	
Perceived severity				0.038 *
Low	1657 (56.3)	706 (42.6)	40.2–45.0	
Moderate	1000 (34.0)	439 (43.9)	40.8–47.0	
High	285 (9.7)	97 (34.0)	28.7–39.7	
Time of the most recent vaccination				<0.001 *
<6 months	255 (8.7)	60 (23.5)	18.6–29.0	
6–12 months	1245 (42.3)	482 (38.7)	36.0–41.4	
12–24 months	1260 (42.8)	586 (46.5)	43.8–49.3	
≥24 months	182 (6.2)	114 (62.6)	55.5–69.4	
Self-reported long COVID				0.031 *
No	792 (26.9)	360 (45.5)	42.0–48.9	
Yes	2150 (73.1)	882 (41.0)	39.0–43.1	
Pandemic fatigue				<0.001 *
Low	1196 (40.7)	290 (24.2)	21.9–26.7	
Moderate	1166 (39.6)	560 (48.0)	45.2–50.9	
High	580 (19.7)	392 (67.6)	63.7–71.3	

* A *p*-value less than 0.05 is considered to be statistically significant; † location” included urban areas (defined as main urban areas, urban–rural junction, and peri-urban areas) and rural areas (defined as townships and villages); “high school and below” included high school and below, technical secondary school, junior college and undergraduate students; master candidates were also included in the “Master’s degree“. For “relationship status”, we divided participants into two categories based on the presence or absence of a lover or cohabiting spouse, with married but separated being considered unpartnered.

**Table 2:** Pandemic fatigue characteristics of 2942 participants recovered from COVID-19 in China.

Characteristics †	Pandemic Fatigue (M ± SD)	*p* Value	Low Pandemic Fatigue	Moderate Pandemic Fatigue	High Pandemic Fatigue	*p* Value
			n (%)	n (%)	n (%)	
Sex		<0.001 *				0.007 *
Male	22.58 ± 9.01		448 (37.8)	475 (40.1)	262 (22.1)	
Female	21.05 ± 8.71		748 (42.6)	691 (39.3)	318 (18.1)	
Age (years)		0.447				0.019 *
<30	21.68 ± 8.68		538 (38.7)	598 (43.0)	254 (18.3)	
30–34	21.93 ± 8.99		324 (41.0)	291 (36.8)	175 (22.2)	
35–39	21.10 ± 9.18		189 (44.9)	149 (35.4)	83 (19.7)	
≥40	21.73 ± 8.93		145 (42.5)	128 (37.5)	68 (19.9)	
Location		0.004 *				0.043 *
Urban	21.81 ± 8.90		1067 (40.1)	1056 (39.7)	539 (20.2)	
Rural	20.28 ± 8.39		129 (46.1)	110 (39.3)	41 (14.6)	
Education		0.160				0.030 *
High school and below	21.26 ± 8.74		306 (40.0)	330 (43.1)	129 (16.9)	
Bachelor’s degree	21.73 ± 8.90		795 (41.2)	744 (38.6)	389 (20.2)	
Master’s degree	22.45 ± 8.93		95 (38.2)	92 (36.9)	62 (24.9)	
Relationship status		0.251				0.545
Without partner	21.31 ± 8.95		258 (41.1)	255 (40.7)	114 (18.2)	
With partner	21.77 ± 8.84		938 (40.5)	911 (39.4)	466 (20.1)	
Smoking		0.002 *				0.060
No	21.45 ± 8.84		1035 (41.5)	979 (39.3)	479 (19.2)	
Yes	22.86 ± 8.89		161 (35.9)	187 (41.6)	101 (22.5)	
Drinking		<0.001 *				0.001 *
No	21.00 ± 8.80		712 (42.9)	658 (39.6)	290 (17.5)	
Yes	22.53 ± 8.88		484 (37.8)	508 (39.6)	290 (22.6)	
Chronic disease		<0.001 *				<0.001 *
No	21.14 ± 8.82		837 (42.8)	780 (39.9)	337 (17.2)	
Yes	22.72 ± 8.85		359 (36.3)	386 (39.1)	243 (24.6)	
Perceived susceptibility		<0.001 *				<0.001 *
Low	21.01 ± 8.97		594 (43.7)	512 (37.7)	252 (18.6)	
Moderate	21.78 ± 8.56		474 (39.4)	509 (42.3)	221 (18.4)	
High	23.67 ± 9.14		128 (33.7)	145 (38.2)	107 (28.2)	
Perceived severity		0.944				0.011*
Low	21.63 ± 9.17		692 (41.8)	616 (37.2)	349 (21.1)	
Moderate	21.69 ± 8.38		385 (38.5)	440 (44.0)	175 (17.5)	
High	21.81 ± 8.73		119 (41.8)	110 (38.6)	56 (19.6)	
Time of the most recent vaccination		<0.001 *				0.001 *
<6 months	19.80 ± 8.99		129 (50.6)	87 (34.1)	39 (15.3)	
6–12 months	21.62 ± 8.74		510 (41.0)	495 (39.8)	240 (19.3)	
12–24 months	21.78 ± 8.87		505 (40.1)	502 (39.8)	253 (20.1)	
≥24 months	23.83 ± 9.03		52 (28.6)	82 (45.1)	48 (26.4)	
Self-reported long COVID		<0.001 *				<0.001 *
No	20.34 ± 9.05		386 (48.7)	266 (33.6)	140 (17.7)	
Yes	22.16 ± 8.75		810 (37.7)	900 (41.9)	440 (20.5)	
Vaccine hesitancy		<0.001 *				<0.001 *
No	19.04 ± 8.17		906 (53.3)	606 (35.6)	188 (11.1)	
Yes	25.26 ± 8.52		290 (23.3)	560 (45.1)	392 (31.6)	

* A *p*-value less than 0.05 is considered to be statistically significant; † “location” included urban areas (defined as main urban areas, urban–rural junctions, and peri-urban areas) and rural areas (defined as townships and villages); “high school and below” included high school and below, technical secondary school, junior college and undergraduate student; master candidates were also included in the “Master’s degree“. For “relationship status”, we divided participants into two categories based on the presence or absence of a lover or cohabiting spouse, with married but separated being considered unpartnered.

**Table 3:** ORs (95%CI) for the hesitancy toward the next dose of COVID-19 vaccines according to pandemic fatigue.

Models	Low Pandemic Fatigue	Moderate Pandemic Fatigue	High Pandemic Fatigue
	OR (95%CI)	OR (95%CI)	OR (95%CI)
Model A	1 (Reference)	2.89 (2.42, 3.44) *	6.51 (5.24, 8.11) *
Model B	1 (Reference)	2.91 (2.44, 3.47) *	6.56 (5.27, 8.17) *
Model C	1 (Reference)	2.96 (2.48, 3.53) *	6.79 (5.44, 8.48) *
Model D	1 (Reference)	2.92 (2.45, 3.49) *	6.80 (5.44, 8.50) *
Model E	1 (Reference)	2.94 (2.46, 3.53) *	6.88 (5.49, 8.64) *

* A *p*-value less than 0.05 is considered to be statistically significant. Model A: unadjusted. Model B: adjusted for sociodemographic factors (gender, age, location, and education). Model C: model B plus health factors (drinking and chronic disease history). Model D: model C plus risk perception factors (perceived susceptibility and perceived severity). Model E: model D plus COVID-19 vaccination history and self-reported long COVID.

## Data Availability

All data in the study are available from the corresponding author by request.

## Supplementary Materials

The following supporting information can be downloaded at: https://www.mdpi.com/article/10.3390/vaccines11101570/s1, Table S1: collection of valid questionnaires by region in mainland China; Table S2: subgroup analysis of the association between pandemic fatigue and COVID-19 vaccine hesitancy among 2942 participants who have recovered from COVID-19 in China.

## References

[1] WHO (2023). COVID-19 Variants. https://www.who.int/activities/tracking-SARS-CoV-2-variants.

[2] WHO (2023). COVID-19 Dashboard. https://www.who.int/emergencies/diseases/novel-coronavirus-2019.

[3] (2023). The Joint Prevention and Control Mechanism of the State Council. Press Conference of the State Council Joint Prevention and Control Mechanism on 8 May 2023. http://www.nhc.gov.cn/xwzb/webcontroller.do?titleSeq=11516&gecstype=1.

[4] Fu D., He G., Li H., Tan H., Ji X., Lin Z., Hu J., Liu T., Xiao J., Liang X. (2023). Effectiveness of COVID-19 Vaccination Against SARS-CoV-2 Omicron Variant Infection and Symptoms—China, December 2022–February 2023. China CDC Wkly..

[5] WHO (2023). Statement on the Fifteenth Meeting of the IHR (2005) Emergency Committee on the COVID-19 Pandemic. https://www.who.int/news/item/05-05-2023-statement-on-the-fifteenth-meeting-of-the-international-health-regulations-(2005)-emergency-committee-regarding-the-coronavirus-disease-(covid-19)-pandemic.

[6] Haynes B.F., Corey L., Fernandes P., Gilbert P.B., Hotez P.J., Rao S., Santos M.R., Schuitemaker H., Watson M., Arvin A. (2020). Prospects for a safe COVID-19 vaccine. Sci. Transl. Med..

[7] Dubé E., Laberge C., Guay M., Bramadat P., Roy R., Bettinger J.A. (2013). Vaccine hesitancy: An overview. Hum. Vaccines Immunother..

[8] Huang Y., Zhang L., Fu J., Wu Y., Wang H., Xiao W., Xin Y., Dai Z., Si M., Chen X. (2023). COVID-19 Vaccine Hesitancy Among Patients Recovered From COVID-19 Infection in Wuhan, China: Cross-Sectional Questionnaire Study. JMIR Public Health Surveill..

[9] WHO (2020). Ten Threats to Global Health in 2019. https://www.who.int/news-room/spotlight/ten-threats-to-global-health-in-2019.

[10] Nuwarda R.F., Ramzan I., Weekes L., Kayser V. (2022). Vaccine Hesitancy: Contemporary Issues and Historical Background. Vaccines.

[11] Letizia A.G., Ge Y., Vangeti S., Goforth C., Weir D.L., Kuzmina N.A., Balinsky C.A., Chen H.W., Ewing D., Soares-Schanoski A. (2021). SARS-CoV-2 seropositivity and subsequent infection risk in healthy young adults: A prospective cohort study. Lancet Respir. Med..

[12] El-Ghitany E.M., Ashour A., Omran E.A., Farghaly A.G., Hassaan M.A., Azzam N.F.A.E.-M. (2022). COVID-19 vaccine acceptance rates and predictors among the Egyptian general population and Healthcare workers, the intersectionality of age and other factors. Sci. Rep..

[13] Nguyen K.H., Huang J., Mansfield K., Corlin L., Allen J.D. (2022). COVID-19 Vaccination Coverage, Behaviors, and Intentions among Adults with Previous Diagnosis, United States. Emerg. Infect. Dis..

[14] Bodas M., Kaim A., Velan B., Ziv A., Jaffe E., Adini B. (2023). Overcoming the effect of pandemic fatigue on vaccine hesitancy—Will belief in science triumph?. J. Nurs. Sch..

[15] Al-Tammemi A.B., Tarhini Z., Akour A. (2021). A swaying between successive pandemic waves and pandemic fatigue: Where does Jordan stand?. Ann. Med. Surg..

[16] Xin L., Wang L., Cao X., Tian Y., Yang Y., Wang K., Kang Z., Zhao M., Feng C., Wang X. (2022). Prevalence and influencing factors of pandemic fatigue among Chinese public in Xi’an city during COVID-19 new normal: A cross-sectional study. Front. Public Health.

[17] Asimakopoulou E., Paoullis P., Shegani A., Argyriadis A., Argyriadi A., Patelarou E., Patelarou A. (2022). Translation, Adaptation and Validation of the Pandemic Fatigue Scale (PFS) in the Greek Language. Healthcare.

[18] Wong L.P., Alias H., Siaw Y.-L., Muslimin M., Lai L.L., Lin Y., Hu Z. (2022). Intention to receive a COVID-19 vaccine booster dose and associated factors in Malaysia. Hum. Vaccines Immunother..

[19] Qin C., Wang R., Tao L., Liu M., Liu J. (2022). Association Between Risk Perception and Acceptance for a Booster Dose of COVID-19 Vaccine to Children Among Child Caregivers in China. Front. Public Health.

[20] Qin C., Yan W., Du M., Liu Q., Tao L., Liu M., Liu J. (2022). Acceptance of the COVID-19 vaccine booster dose and associated factors among the elderly in China based on the health belief model (HBM): A national cross-sectional study. Front. Public Health.

[21] Daud S. (2021). The COVID-19 Pandemic Crisis in Malaysia and the Social Protection Program. J. Dev. Soc..

[22] (2023). Star Survey. Sample Service. https://www.wjx.cn/sample/service.aspx.

[23] Qin C., Du M., Wang Y., Liu Q., Yan W., Tao L., Liu M., Liu J. (2023). Assessing acceptability of the fourth dose against COVID-19 among Chinese adults: A population-based survey. Hum. Vaccines Immunother..

[24] Lilleholt L., Zettler I., Betsch C., Böhm R. (2020). Pandemic fatigue: Measurement, correlates, and consequences. PsyArXiv.

[25] Marsella A.J., Leong F.T.L. (1995). Cross-Cultural Issues in Personality and Career Assessment. J. Career Assess..

[26] Janz N.K., Becker M.H. (1984). The Health Belief Model: A Decade Later. Health Educ. Q..

[27] Qin C., Wang R., Tao L., Liu M., Liu J. (2022). Acceptance of a Third Dose of COVID-19 Vaccine and Associated Factors in China Based on Health Belief Model: A National Cross-Sectional Study. Vaccines.

[28] Baccolini V., Renzi E., Isonne C., Migliara G., Massimi A., De Vito C., Marzuillo C., Villari P. (2021). COVID-19 Vaccine Hesitancy among Italian University Students: A Cross-Sectional Survey during the First Months of the Vaccination Campaign. Vaccines.

[29] Liu Y., Han J., Li X., Chen D., Zhao X., Qiu Y., Zhang L., Xiao J., Li B., Zhao H. (2021). COVID-19 Vaccination in People Living with HIV (PLWH) in China: A Cross Sectional Study of Vaccine Hesitancy, Safety, and Immunogenicity. Vaccines.

[30] Hong J., Xu X.-W., Yang J., Zheng J., Dai S.-M., Zhou J., Zhang Q.-M., Ruan Y., Ling C.-Q. (2022). Knowledge about, attitude and acceptance towards, and predictors of intention to receive the COVID-19 vaccine among cancer patients in Eastern China: A cross-sectional survey. J. Integr. Med..

[31] Hu S., Liu J., Li S., Wu Q., Wang X., Xu D., Chen Y. (2023). Patients with IBD have a more cautious attitude towards COVID-19 vaccination. Front. Immunol..

[32] Qin C., Yan W., Tao L., Liu M., Liu J. (2022). The Association between Risk Perception and Hesitancy toward the Booster Dose of COVID-19 Vaccine among People Aged 60 Years and Older in China. Vaccines.

[33] Gerussi V., Peghin M., Palese A., Bressan V., Visintini E., Bontempo G., Graziano E., De Martino M., Isola M., Tascini C. (2021). Vaccine Hesitancy among Italian Patients Recovered from COVID-19 Infection towards Influenza and Sars-Cov-2 Vaccination. Vaccines.

[34] Petravić L., Arh R., Gabrovec T., Jazbec L., Rupčić N., Starešinič N., Zorman L., Pretnar A., Srakar A., Zwitter M. (2021). Factors Affecting Attitudes towards COVID-19 Vaccination: An Online Survey in Slovenia. Vaccines.

[35] Wu T., Jia X., Shi H., Niu J., Yin X., Xie J., Wang X. (2021). Prevalence of mental health problems during the COVID-19 pandemic: A systematic review and meta-analysis. J. Affect. Disord..

[36] Yue Y., Li L., Liu R., Zhang Y., Zhang S., Sang H., Tang M., Zou T., Shah S.M., Shen X. (2023). The dynamic changes of psychosomatic symptoms in three waves of COVID-19 outbreak and fatigue caused by enduring pandemic in China. J. Affect. Disord..

[37] Ali-Saleh O., Bord S., Basis F. (2022). Factors Associated with Decisions of Arab Minority Parents in Israel to Vaccinate Their Children against COVID-19. Vaccines.

[38] WHO (2020). Pandemic Fatigue: Reinvigorating the Public to Prevent COVID-19. https://apps.who.int/iris/bitstream/handle/10665/335820/WHO-EURO-2020-1160-40906-55390-eng.pdf.

[39] Reicher S., Drury J. (2021). Pandemic fatigue? How adherence to covid-19 regulations has been misrepresented and why it matters. BMJ.

