# Supplementary material for: Pandemic Fatigue and Vaccine Hesitancy among People Who Have Recovered from COVID-19 Infection in the Post-Pandemic Era: Cross-Sectional Study in China
Source: Vaccines (Basel). 2023 Oct 5;11(10):1570. doi: 10.3390/vaccines11101570 (PMC10610579; doi:10.3390/vaccines11101570)
Supplement: Supplementary file 1 [file vaccines-11-01570-s001.zip › vaccines-2600044-supplementary.pdf]

Supplemental File

Table S1. Collection of valid questionnaires by region in mainland China.

Regions	Number	Proportion(%)
Anhui Province	130	4.42
Beijing	47	1.60
Chongqing	66	2.24
Fujian Province	86	2.92
Gansu Province	48	1.63
Guangdong Province	266	9.04
Guangxi Province	106	3.60
Guizhou Province	82	2.79
Hainan Province	21	0.71
Hebei Province	158	5.37
Heilongjiang Province	65	2.21
Henan Province	208	7.07
Hubei Province	123	4.18
Hunan Province	140	4.76
Jiangsu Province	180	6.12
Jiangxi Province	95	3.23
Jilin Province	51	1.73
Liaoning Province	91	3.09
Ningxia Province	14	0.48
Qinghai Province	11	0.37
Shaanxi Province	84	2.86
Shandong Province	214	7.27
Shanghai	53	1.80
Shanxi Province	74	2.52
Sichuan Province	177	6.02
Nei Monggol Autonomous Region	50	1.70
Xinjiang Uygur Autonomous Region	53	1.80
Tianjin	30	1.02
Tibet	7	0.24
Yunnan Province	75	2.55
Zhejiang Province	137	4.66

Table S2. Subgroup analysis of the association between pandemic fatigue and COVID-19 vaccine hesitancy among 2942 participants recovered from COVID-19 in China.

**Characteristics†**	**Low pandemic fatigue**	**Moderate pandemic fatigue**	**High pandemic fatigue**	**P for interaction**
	**OR (95%CI)**	**OR (95%CI)**	**OR (95%CI)**	
Sex				0.750
Male	Reference	2.99 (2.24, 3.99)	7.00 (4.93, 9.95)	
Female	Reference	2.97 (2.35, 3.75)	7.04 (5.20, 9.53)	
Age (years)				0.263
<30	Reference	2.69 (2.07, 3.49)	5.42 (3.87, 7.60)	
30-34	Reference	3.83 (2.64, 5.56)	10.96 (6.98, 17.20)	
35-39	Reference	3.34 (2.02, 5.52)	9.73 (5.13, 18.44)	
≥40	Reference	2.80 (1.63, 4.81)	7.59 (3.77, 15.30)	
Location				0.423
Urban	Reference	3.04 (2.51, 3.68)	7.24 (5.70, 9.19)	
Rural	Reference	2.43 (1.36, 4.33)	5.75 (2.49, 13.26)	
Education				0.037*
High school and below	Reference	2.91 (2.04, 4.16)	10.97 (6.61, 18.22)	
Bachelor’s degree	Reference	3.12 (2.49, 3.92)	7.02 (5.30, 9.30)	
Master’s degree	Reference	2.49 (1.28, 4.85)	3.88 (1.84, 8.17)	
Relationship status				0.645
Without partner	Reference	3.15 (2.13, 4.65)	7.55 (4.44, 12.83)	
With partner	Reference	2.92 (2.37, 3.60)	6.87 (5.33, 8.87)	
Smoking				<0.001*
No	Reference	2.94 (2.42, 3.57)	6.60 (5.15, 8.45)	
Yes	Reference	3.57 (2.09, 6.09)	10.47 (5.60, 19.57)	
Drinking				0.513
No	Reference	3.24 (2.56, 4.12)	6.59 (4.82, 9.01)	
Yes	Reference	2.59 (1.95, 3.45)	7.26 (5.18, 10.18)	
Chronic disease				0.445
No	Reference	2.87 (2.31, 3.56)	7.05 (5.28, 9.41)	
Yes	Reference	3.10 (2.23, 4.33)	6.94 (4.74, 10.17)	
Perceived susceptibility				0.704
Low	Reference	2.87 (2.20, 3.74)	7.10 (5.06, 9.97)	
Moderate	Reference	3.14 (2.36, 4.17)	7.47 (5.16, 10.82)	
High	Reference	2.53 (1.47, 4.38)	5.95 (3.26, 10.86)	
Perceived severity				0.500
Low	Reference	3.06 (2.40, 3.90)	7.47 (5.54, 10.07)	

Moderate	Reference	3.11 (2.28, 4.25)	8.09 (5.32, 12.30)	
High	Reference	2.00 (1.08, 3.69)	3.78 (1.77, 8.08)	
Time of the most recent vaccination				0.625
<6 months	Reference	2.94 (1.38, 6.29)	8.11 (3.25, 20.25)	
6-12 months	Reference	2.76 (2.08, 3.65)	6.95 (4.91, 9.85)	
12-24 months	Reference	3.19 (2.44, 4.18)	6.77 (4.80, 9.56)	
≥24 months	Reference	2.67 (1.18, 6.04)	11.14 (3.63, 34.20)	
Self-reported long COVID				0.427
No	Reference	2.45 (1.75, 3.43)	5.14 (3.32, 7.97)	
Yes	Reference	3.20 (2.57, 3.98)	8.03 (6.13, 10.53)	

* A p-value less than 0.05 is considered to be statistically significant.

† "Location" included urban areas (defined as main urban areas, urban–rural junction and peri-urban areas) and rural areas (defined as townships and villages); "high school and below" included high school and below, technical secondary school, junior college and undergraduate student; master candidates were also included in the "Master's degree". For "relationship status", we divided participants into two categories based on the presence or absence of a lover or cohabiting spouse, with married but separated being considered unpartnered.
